# Antimicrobial activity of piperacillin/tazobactam against key peri-implant pathogens: an *in vitro* comparative study with amoxicillin-clavulanate and minocycline

**DOI:** 10.4317/medoral.27797

**Published:** 2025-10-14

**Authors:** Dolores Hurtado-Celotti, María Andrés-Veiga, Cristina Madrigal Martínez-Pereda, Cristina Meniz-García, Juan Santos-Marino, Natalia Martínez-Rodríguez

**Affiliations:** 1 Department of Dental Clinical Specialties, Faculty of Dentistry, Complutense University of Madrid, Spain; 2 Alfonso X El Sabio University, Madrid, Spain; 3 Surgical and Implant Therapies in the Oral Cavity Research Group, Complutense University of Madrid, Spain; 4 Department of Surgery. Faculty of Medicine. University of Salamanca, Spain

## Abstract

**Background:**

Peri-implantitis is an inflammatory disease linked to bacterial biofilms that threatens the long-term success of dental implants. The growing problem of antibiotic resistance among peri-implant pathogens highlights the need to explore alternative antimicrobial agents with proven *in vitro* efficacy.

**Material and Methods:**

This *in vitro* study evaluated the antimicrobial activity of piperacillin/tazobactam compared with two commonly used antibiotics in dentistry: amoxicillin-clavulanate and minocycline. The minimum inhibitory concentrations (MICs) were determined using Etest® gradient diffusion strips against three key peri-implant pathogens: *Porphyromonas *gingivalis**, *Prevotella intermedia*, and *Aggregatibacter actinomycetemcomitans*. Cultures were incubated under anaerobic conditions to simulate the peri-implant environment.

**Results:**

Piperacillin/tazobactam demonstrated MIC values comparable to those of amoxicillin-clavulanate and minocycline for *P. *gingivalis** and *A. actinomycetemcomitans*, and lower MICs against *P. intermedia*. Statistical analysis confirmed that piperacillin/tazobactam is not inferior to these widely used antibiotics.

**Conclusions:**

Piperacillin/tazobactam shows promising *in vitro* antimicrobial activity against key peri-implant pathogens and may serve as an effective alternative or adjunctive treatment in managing peri-implantitis. Further clinical studies are warranted to confirm its efficacy and safety *in vivo*.

** Key words:**Peri-implantitis, piperacillin/tazobactam, antimicrobial resistance, in vitro study, oral microbiology.

## Introduction

Currently, the treatment of peri-implantitis poses a challenge in the field of dentistry due to the increase, in recent decades, of implant-based rehabilitations. Several treatment options have been proposed, including non-surgical therapies such as mechanical debridement; pharmacological therapy with chlorhexidine irrigation or local antibiotics, and the administration of systemic antibiotics; surgical procedures involving the elevation of a flap to remove bacteria, smoothing the implant surface, and decontaminating it using chemical agents or lasers [1]. In some cases, it may be necessary to correct anatomical conditions by eliminating pathological peri-implant pockets to improve plaque control and prevent a favorable environment for bacterial colonization. This can be achieved through resective procedures or guided bone regeneration techniques, using autogenous or allogeneic bone grafts [2].

Studies comparing the microbiota in peri-implant and periodontal disease have found greater bacterial diversity in peri-implantitis compared to periodontal samples from the same patients, with the *Porphyromonas* and Treponema species being more prevalent in peri-implantitis. In contrast, *Aggregatibacter* species are more commonly found in periodontal lesions. Therefore, both oral conditions can be considered different yet related diseases [3]. These findings align with a meta-analysis by Carvalho *et al*. [4], which associated peri-implantitis with the presence of *Staphylococcus epidermidis* and specific periodontal pathogens: *Porphyromonas *gingivalis* (Pg)*,*Prevotella intermedia (Pi)*, **Tannerella forsythia* (Tf)*, *Treponema denticola (Td)*, and **Fusobacterium nucleatum* (Fn)*. Even after treatment with both surgical and non-surgical approaches, bacteria such as Pi, Fn, and *Peptostreptococcus micros* tend to persist, indicating early recolonization. One month after treatment, the count of *Pi* increases. Conversely, the count of *Aggregatibacter actinomycetemcomitans* (Aa) generally decreases for approximately three months [5].

Several local antimicrobials have been used, including tetracycline fibers, doxycycline gel, and minocycline microspheres. The adjunctive use of slow-release doxycycline was evaluated in a controlled study where the prosthetic suprastructure was removed prior to non-surgical therapy, followed by mechanical cleaning and irrigation with 0.2% chlorhexidine. The study concluded that topical application of this antimicrobial significantly improved outcomes. Furthermore, in a series of randomized controlled trials, clinical improvement was observed, including reduced bleeding on probing and probing depth [6-10].

Current studies are exploring the combination of resective and regenerative surgical therapy using allogeneic materials impregnated with vancomycin and tobramycin, showing promising results despite limited follow-up periods [11].

Other antibiotics are emerging as alternatives for the treatment of peri-implantitis in light of increasing bacterial resistance. One such antibiotic is piperacillin, a beta-lactam antibiotic combined with tazobactam, a beta-lactamase inhibitor. This combination offers a broad spectrum of activity against Gram-positive and Gram-negative pathogens, as well as both aerobic and anaerobic organisms. Its effectiveness has been studied in patients with stage III and IV periodontitis in various studies. Hurtado-Celotti *et al*. [12], applied it topically as an adjunct to scaling and root planing compared to conventional treatment over a 6-month follow-up. A greater reduction in clinical attachment level, probing depth, and plaque index was observed in the test group. Microbiologically, its application also resulted in a significant reduction in periodontal pathogens. However, these results were not maintained long-term, indicating the need for recurrent administration.

Ilyes *et al*. [13] compared the use of piperacillin/tazobactam to doxycycline gel and a control group after mechanical instrumentation. The piperacillin/tazobactam group showed a slightly greater reduction in probing depth, although the difference was not statistically significant compared to the other two groups. The same authors evaluated the clinical effect of systemic amoxicillin + metronidazole for 7 days versus local application of piperacillin/tazobactam (both following subgingival mechanical instrumentation). Both treatment regimens showed similar outcomes after three months in terms of probing depth, clinical attachment level, bleeding on probing, and reduction in the presence of Aa, Pg, Pi, Tf, and *Td* bacteria [14]. In patients with peri-implant mucositis, adjunctive treatment with piperacillin/tazobactam gel resulted in a greater reduction in bleeding on probing compared to conventional therapy, although no significant differences were observed in other clinical and microbiological parameters [15].

To date, only one article in the literature has evaluated the combined efficacy of piperacillin-tazobactam with implantoplasty in 43 patients with peri-implantitis. A significant reduction was observed in probing depth, bleeding, and suppuration on probing at one year of follow-up. Additionally, average bone regeneration of 2.64 mm ± 1.59 (*p*<0.001) was achieved in the defect area [16].

Currently, the scientific evidence available regarding the use of piperacillin-tazobactam in periodontal and peri-implant diseases is limited. The objective of this study is to evaluate *in vitro* the inhibitory efficacy of this drug on bacteria associated with peri-implantitis, compared to other broad-spectrum antibiotics such as amoxicillin-clavulanic acid and minocycline.

## Material and Methods

- Study design

An *in vitro* study was conducted to compare the inhibitory efficacy of the antibiotics: amoxicillin-clavulanic acid (AMX), minocycline (MC), and piperacillin/tazobactam (PTZ), against the microorganisms Aa, Pg, and Pi.

- Materials

Culture plates: Prepared with agar medium (OxoidLimited, CM00067, Thermo Fisher Scientific, UK) supplemented with 5% defibrinated horse blood (Oxoid, SR0050, Thermo Fisher Scientific, UK) and 5% Hemin-Menadione solution (12.2 mL per liter, respectively) (Merck, Spain).

Bacterial strains: Obtained from the American Type Culture Collection (ATCC) for Pg (ATCC 33277), Pi, and *Aa* (DSMZ 8324), and cultured in Brain Heart Infusion (BHI) medium (Becton, Dickinson and Company, Franklin Lakes, NJ, USA).

Antibiotics: All three antibiotics were acquired as gradient diffusion strips marked with MIC values ranging from 256 to 0.016 μg/mL (Etest®, BioMérieux).

Applicators: Application of the Etest® strips to the culture plates was performed using a Mini Grip-It® suction applicator (BioMérieux, ref. 411200), along with sterile cotton swabs (Deltalab, Spain, ref. 300200) to remove air bubbles and ensure optimal strip-medium contact.

Microscope: A stereoscopic magnifying lens (Kyowa model SD-2PL; Twin HWF10x) was used to visualize the inhibition halos generated by the Etest® strips and assess antibiotic inhibition.

Spectrophotometer: Various spectrophotometric measurements were taken before bacterial culturing to ensure appropriate colony-forming unit (CFU) counts (Shimadzu UV-1800 spectrophotometer).

- Methods

The different phases of the process were as follows:

Strain recovery: Bacterial strains (Aa, Pg, Pi) were obtained using cryobeads and inoculated on culture media. Plates were incubated under anaerobic conditions at 37°C for 3 days to promote bacterial growth. Afterward, the bacteria were suspended in BHIA solutions for subsequent plating on blood agar plates.

Spectrophotometric analysis: BHIA suspensions were analyzed using McFarland standards to quantify CFUs per millilitre. AccepTable ranges were established between 0 and 1.

Culturing and strip application: After verifying the spectrophotometric results, each bacterium (Aa, Pg, Pi) was cultured on agar plates (*n*=90). A volume of 100 μL of each BHIA bacterial suspension was applied per plate and spread using sterile swabs. Once the suspension was evenly spread, plates were left to stand for 10 ± 2 minutes to allow fixation. Then, the Etest® strips were placed under aseptic conditions using the Mini Grip-It applicator. Ten strips each of AMX, MC, and PTZ were applied per microorganism. The plates were incubated for 3-5 days, depending on the microorganism, under anaerobic conditions at 37°C, prior to evaluation of bacterial growth and inhibition.

- Evaluation of microbiological data

**Figure 1:**
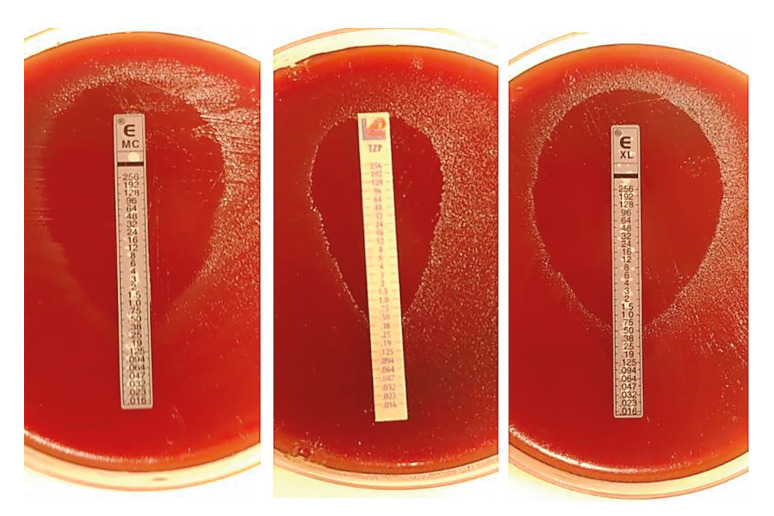
Observation of inhibition halos under a stereoscopic lens to determine the Minimum Inhibitory Concentration (MIC).

Microscopic evaluation of the Minimum Inhibitory Concentration (MIC) for each antibiotic was performed. Plates were examined under the microscope to better observe inhibitory zones. The inhibition halo for each strip was recorded, defining the limit as the area adjacent to the strip where no bacterial growth was observed. In unclear cases, the next lowest inhibition value was chosen, according to manufacturer specifications (Fig. 1).

- Statistical analysis

A two-factor analysis of variance (ANOVA) with interaction was used to compare the mean values. The two main factors were the antibiotic used and the target microorganism, along with their interaction.

To determine statistically significant differences in means—and thereby assess the differing effects of antibiotics on the bacteria—ANOVA variance decomposition and Levene’s test for equality of variances were employed, followed by pairwise comparisons of levels for each factor. The significance level was set at *p* < 0.05 with a 95% confidence interval.

## Results

**Figure 2:**
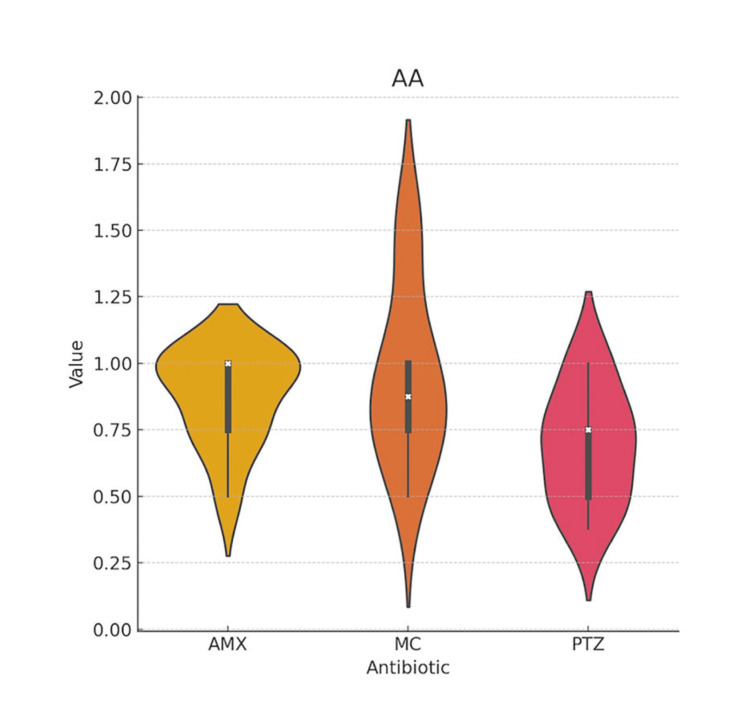
Violin plot of the minimum inhibitory concentrations (MIC) of amoxicillin-clavulanic acid (AMX), minocycline (MC), and piperacillin/tazobactam (PTZ) against <italic>Aggregatibacter actinomycetemcomitans</italic> (AA).

**Figure 3:**
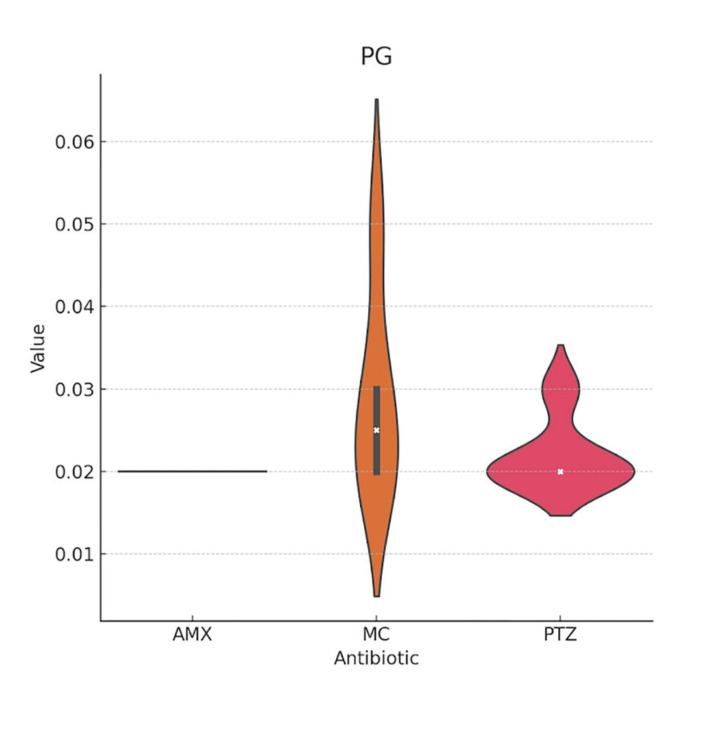
Violin plot of the minimum inhibitory concentrations (MIC) of amoxicillin-clavulanic acid (AMX), minocycline (MC), and piperacillin/tazobactam (PTZ) against <italic>Porphyromonas *gingivalis*</italic> (PG).

**Figure 4:**
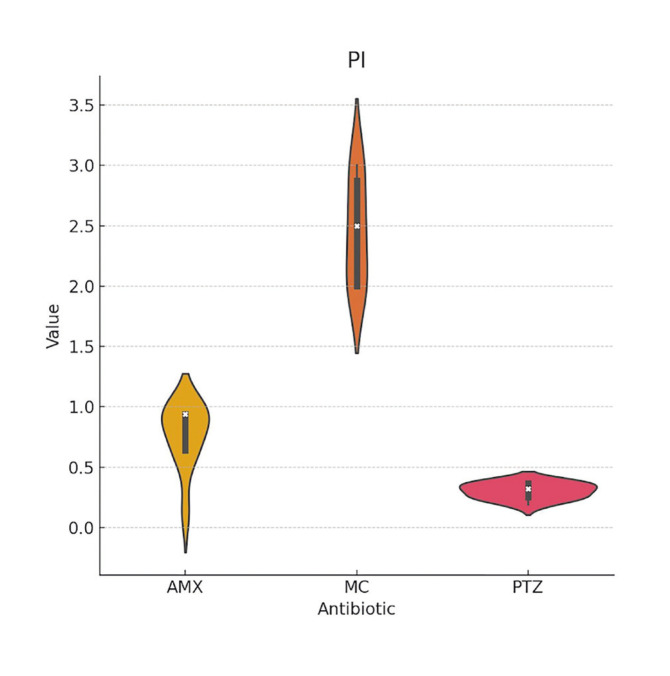
Violin plot of the minimum inhibitory concentrations (MIC) of amoxicillin-clavulanic acid (AMX), minocycline (MC), and piperacillin/tazobactam (PTZ) against <italic>Prevotella intermedia</italic> (PI).

The mean MIC values found for *Aa* were slightly lower for PTZ compared to AMX and MC; however, these differences were not statistically significant (Table 1, Fig. 2). The response of Pg to the antibiotics was very similar between AMX and PTZ, and slightly better with MC (Table 2, Fig. 3). In the case of Pi, PTZ required the lowest inhibitory concentration, followed by AMX, with these differences being statistically significant (Table 3, Fig. 4).

## Discussion

Dental implants are a safe and long-term functional alternative, which is why their use has become increasingly widespread in the rehabilitation of partially and completely edentulous patients. However, the number of implants placed is directly proportional to the prevalence of peri-implant inflammatory complications [17].

The literature suggests that the microbiological etiology of peri-implantitis is very similar to that of severe periodontitis (Aa, Pg, Pi, Tf, and Td), and that the chemical composition and surface of the implant have a significant impact on the bacterial biofilm that forms. This biofilm is the main cause of peri-implantitis development, and therefore, one of the goals of peri-implant therapy is its elimination, both surgically and non-surgically. For this reason, the study of antibiotic therapies becomes particularly important when combined with mechanical debridement [18].

To date, the literature comparing the clinical efficacy of different antibiotics in the treatment of peri-implantitis is very limited. However, there are studies evaluating the efficacy of antibiotics such as doxycycline compared to mechanical debridement alone [19]. Other studies, such as that by Renvert *et al*. [8], compared the subgingival placement of minocycline spheres (1 mg and 3 mg) with a 1% chlorhexidine antiseptic gel, both combined with mechanical debridement. The antibiotic group showed better outcomes, with significant reductions in probing depth and bleeding. More recently, Mensi *et al*. [20], developed a minimally invasive protocol combining mechanical debridement, topical doxycycline application, and air polishing (MAINST) in a series of 15 cases. Statistically significant clinical reductions in bleeding on probing and probing depth were achieved.

Another antibiotic similar to doxycycline, minocycline, was studied by Schär *et al*. [21], who conducted a study on 40 implants comparing photodynamic therapy with topical minocycline application. Both groups showed similar results, with a significant reduction in probing depth and gingival recession after 3 months. These values remained sTable at 6 and 12 months, and mucosal inflammation was permanently resolved in 15% of cases, suggesting that minocycline may be used as an adjunctive agent in peri-implantitis therapy, combined with mechanical debridement.

Vancomycin and tobramycin have been studied for peri-implantitis treatment in combination with allogeneic bone regeneration materials, referring to surgical therapies. Tetracycline, amoxicillin, and metronidazole have also been explored (11). Regarding tetracycline, Mombelli *et al*. [22], conducted a study on 30 implants with varying degrees of bone loss and peri-implantitis, where tetracycline hydrochloride polymer fibers were inserted subgingivally into the defects and removed after 10 days. The study reported clinical improvements in bleeding on probing and pocket depth reduction. Although a significant reduction in the number of microorganisms—particularly Pi—was initially observed, the counts of *Aa* and Pg did not significantly change by the end of the study. These results contrast with those of the current study, which showed lower inhibitory efficacy of minocycline against *Pi* and similar values for *Aa* and Pg. This discrepancy may be due to the sustained release over 10 days in Mombelli's study and individual oral environments.

Broad-spectrum antibiotics such as amoxicillin have generally been evaluated for peri-implantitis treatment through systemic administration alongside mechanical debridement and surgical access, as in the study by Heitz-Mayfield *et al*. [23]. However, there is limited scientific evidence regarding the topical adjunctive use of amoxicillin or amoxicillin-clavulanic acid. Given their broad-spectrum nature, these antibiotics may offer non-inferior results compared to agents such as tetracyclines, minocycline, and doxycycline.

Antibiotic resistance has been assessed by Ardila *et al*. [24,25], in both periodontitis and peri-implantitis patients. For periodontal disease, Aa, Tf, and Pg showed low resistance to amoxicillin, but high resistance to tetracycline, metronidazole, and azithromycin. In peri-implant pathology, Pg and *Fn* showed high resistance to tetracycline, metronidazole, and erythromycin, and lesser resistance to clindamycin. *Pi* and *Aa* also showed high resistance to erythromycin. The latter was highly resistant to both clindamycin and doxycycline. Notably, 72% of the 120 patients included in the study had submucosal microorganisms resistant to one or more of the tested antibiotics.

The use of antibiotics (amoxicillin + metronidazole or phenoxymethylpenicillin + metronidazole) as an adjunct to surgical treatment of peri-implantitis has shown better results in probing depth and marginal bone stability compared to resective surgery and placebo. This was described by Riben *et al*. [26], in a trial involving 113 implants. All groups showed clinical and radiological improvement over time, but statistically significant differences in marginal bone stability were observed: 97% for the amoxicillin + metronidazole group, 89% for the phenoxymethylpenicillin + metronidazole group, and 76% for the placebo group. Levels of *Aa* and *Tf* were lower in the antibiotic groups.

Currently, there is insufficient evidence to support a specific, evidence-based antibiotic protocol for the treatment of peri-implantitis [27]. However, it is clear that the administration of this class of drugs leads to improvements in various clinical parameters.

Although this study provides valuable insights into the *in vitro* efficacy of piperacillin/tazobactam against key peri-implant pathogens, several limitations must be acknowledged. First, the antimicrobial activity was evaluated exclusively under controlled laboratory conditions using isolated bacterial strains. While Etest® methodology is a standardized and widely accepted technique for determining minimum inhibitory concentrations (MICs), it does not replicate the complex *in vivo* environment of the oral cavity, where factors such as saliva, immune responses, and the structure of biofilms influence antimicrobial effectiveness.

Second, the study used reference strains from culture collections rather than clinical isolates obtained from patients with peri-implantitis. Clinical strains often exhibit greater variability in resistance profiles, and future studies should incorporate patient-derived samples to enhance the clinical relevance of the findings.

Third, no assessment was made of the pharmacokinetics or tissue penetration of piperacillin/tazobactam in the peri-implant environment. Even if MIC values are low, effective concentrations must be achieved at the target site, particularly when using topical formulations.

Fourth, the study focused on three bacterial species commonly associated with peri-implantitis; however, the peri-implant microbiome is polymicrobial and dynamic. Other relevant microorganisms —including Fn, Tf, or opportunistic pathogens like *Staphylococcus epidermidis*— were not included.

Finally, the long-term effect of piperacillin/tazobactam, including the potential for recolonization and resistance development after repeated use, was not evaluated. These aspects should be addressed in future *in vivo* and clinical studies to assess therapeutic outcomes and safety over time.

Considering these limitations, while the *in vitro* results are promising, caution must be exercised when extrapolating them to clinical applications. Further research, including randomized controlled clinical trials and biofilm model studies, is needed to validate the use of piperacillin/tazobactam as a viable adjunct in the management of peri-implantitis.

## Conclusions

The results of this *in vitro* study demonstrate that the antibiotic combination piperacillin/tazobactam exhibits antimicrobial activity comparable to, and in some cases superior to, that of amoxicillin-clavulanate and minocycline against key peri-implantitis-associated pathogens, including *Prevotella intermedia*, *Porphyromonas *gingivalis**, and *Aggregatibacter actinomycetemcomitans*. In particular, the lower minimum inhibitory concentration observed against *P. intermedia* highlights the potential of this combination as a promising therapeutic agent or adjunct in the management of peri-implantitis. Given the growing challenge of antimicrobial resistance in the treatment of oral infectious diseases, our findings support the continued investigation of piperacillin/tazobactam, especially in topical formulations, as part of an integrated treatment approach alongside mechanical debridement. However, further clinical research, including *in vivo* studies and randomized controlled trials, is needed to confirm its therapeutic efficacy, safety profile, and practical application in real-world settings.

## Figures and Tables

**Table 1:** Mean values, medians, and standard deviations of the minimum inhibitory concentrations for * Aggregatibacter actinomycetemcomitans*.

Bacteria	Antibiotic	Valid N	Mean	Standard Deviation	Median	Percentile 25	Percentile 75	Min.	Max.
Aa	AMX	10	0.88	0.18	1.00	0.75	1.00	0.50	1.00
	MC	10	0.85	0.27	0.75	0.75	1.00	0.50	1.50
	PTZ	10	0.71	0.20	0.75	0.50	0.75	0.38	1.00
	Total	30	0.81	0.22	0.75	0.75	1.00	0.38	1.50

Aa: Aggregatibacter actinomycetemcomitans; AMX: amoxicillin-clavulanic acid; MC: minocycline; PTZ: piperacillin/tazobactam.

**Table 2:** Mean values, medians, and standard deviations of the minimum inhibitory concentrations for *Porphyromonas gingivalis*.

Bacteria	Antibiotic	Valid N	Mean	Standard Deviation	Median	Percentile 25	Percentile 75	Min.	Max.
Pg	AMX	10	0.02	0.00	0.02	0.02	0.02	0.02	0.02
	MC	10	0.03	0.01	0.02	0.02	0.03	0.02	0.05
	PTZ	10	0.02	0.00	0.02	0.02	0.02	0.02	0.03
	Total	30	0.02	0.01	0.02	0.02	0.02	0.02	0.05

Pg: Porphyromonas gingivalis; AMX: amoxicillin-clavulanic acid; MC: minocycline; PTZ: piperacillin/tazobactam.

**Table 3:** Mean values, medians, and standard deviations of the minimum inhibitory concentrations for * Prevotella intermedia*.

Bacteria	Antibiotic	Valid N	Mean	Standard Deviation	Median	Percentile 25	Percentile 75	Min.	Max.
Pi	AMX	10	0.75	0.34	0.94	0.64	0.94	0.13	0.94
	MC	10	2.50	0.53	2.50	2.00	3.00	2.00	3.00
	PTZ	10	0.31	0.08	0.32	0.25	0.38	0.19	0.38
	Total	30	1.19	1.03	0.94	0.38	2.00	0.13	3.00

Pi: Prevotella intermedia; AMX: amoxicillin-clavulanic acid; MC: minocycline; PTZ: piperacillin/tazobactam.
